# Core Interpersonal Patterns in Complex Trauma and the Process of Change in Psychodynamic Therapy: A Case Comparison Study

**DOI:** 10.3389/fpsyg.2020.00122

**Published:** 2020-02-11

**Authors:** Kimberly Van Nieuwenhove, Reitske Meganck

**Affiliations:** Ghent University, Ghent, Belgium

**Keywords:** case comparison study, systematic case study, childhood trauma, interpersonal patterns, core conflictual relationship theme, treatment process

## Abstract

We performed a case comparison study to investigate the nature of interpersonal patterns in childhood trauma and the process of change therein. We analyzed three matching cases of childhood trauma that followed a psychodynamic treatment *via* a mixed-methods design. We found that (1) the core tendency to avoid negative reactions from others through passive behaviors emerged in all three cases, both in childhood and adulthood, (2) core interpersonal patterns transpired in the interaction between patient and therapist and thereby affected the therapeutic relationship, and (3) change ensued when a repetition of core interpersonal patterns was avoided and a new relational experience occurred. The accumulated findings across cases further resulted in several clinical implications and recommendations, such as the importance of the assessment of patients’ (covert) conditions, responsiveness, supervision and facilitating patients’ agency, and provided several avenues for further research.

A systematic review conducted by Van Nieuwenhove and Meganck (2017) revealed the importance of interpersonal features in childhood trauma on three levels, namely etiology, consequences, and treatment. Unfortunately, the available literature up until now only provides a static examination of interpersonal difficulties experienced by patients with a childhood trauma background and yields inconsistent findings regarding dominant interpersonal patterns (Van Nieuwenhove and Meganck, 2017). Therefore, a more thorough investigation of the nature of interpersonal patterns in childhood trauma is warranted. Several studies have used the Core Conflictual Relationship Theme (CCRT) method (Luborsky and Crits-Christoph, 1998) to study interpersonal patterns in cases with a traumatic background (e.g., Okey et al., 2000; Drapeau and Perry, 2009). The CCRT operationalizes interpersonal patterns by defining the main wish (W), the main response of the other (RO), and the self (RS) in narrative material (e.g., interview or therapy excerpts) concerning very specific relational encounters – i.e., relationship episodes (RE). However, these studies are mainly based on small-scale cross-sectional samples, which does not allow for an in-depth understanding of the dynamic and complex nature of interpersonal relationship patterns. Moreover, these studies do not allow an understanding of *how* interpersonal patterns transpire in a therapeutic context and *how* they influence the therapeutic process.

On the level of treatment, researchers argue that a safe therapeutic alliance is difficult to establish with patients with a childhood trauma background because of their overall difficulty in trusting others (e.g., Pearlman and Courtois, 2005). Further, it is argued that these trust issues complicate the revision and reworking of interpersonal difficulties in treatment (e.g., Lawson et al., 2013). Therefore, it is warranted that additional and explicit attention is paid to the formation of a safe therapeutic relationship early in treatment (e.g., Gleiser et al., 2008). Although these issues have been well-endorsed theoretically, the formation of the therapeutic relationship with patients with a childhood trauma background and the process of change in treatment has hardly ever been studied in a systematic way (Van Nieuwenhove and Meganck, 2017).

The conclusion to be drawn from these observations is that several questions remain unanswered in the field of trauma studies, especially with regards to the nature of interpersonal patterns and the way treatment can address the interpersonal difficulties associated with childhood trauma. Therefore, we put forward the following research questions: (1) what is the specific nature of interpersonal patterns in childhood trauma?; (2) how do interpersonal patterns change throughout treatment?; (3) how is a safe therapeutic relationship established in treatment?; and (4) which interventions are used to address interpersonal problems in treatment?

In order to answer these questions, we examined interpersonal features in three systematic case studies of patients with a background of childhood trauma who received supportive-expressive psychodynamic therapy, namely in the case of James (pseudonym) (Van Nieuwenhove et al., 2018), Amy (pseudonym) (Van Nieuwenhove et al., 2020), and Pam (pseudonym) (Van Nieuwenhove et al., unpublished). In this study, we provide a cross-case comparison to integrate and discuss the main findings derived from the three separate cases. The analytic process consists of systematically identifying commonalities and dissimilarities between cases and provides alternative interpretations by identifying underlying mechanisms that might explain the convergent and divergent findings (Iwakabe and Gazzola, 2009).

## Methods

The case comparison method consists of systematically comparing two or more cases of patients with similar problems who are treated in similar conditions (e.g., Watson et al., 2007). Because client characteristics and the treatment conditions are equivalent in many regards, this approach allows for the identification of cross-case similarities and dissimilarities and enables the examination of common core processes across cases and the exploration of possible alternative explanations for unique or distinctive features within individual cases (e.g., Iwakabe and Gazzola, 2009).

### Participants

#### Patients

James^1^, a White male, who works a blue-collar job in sales, was 23 years old when he entered therapy. During his childhood, his father was both verbally and physically aggressive toward him and his brothers, while his mother remained a passive witness. James met the criteria for the diagnosis of Posttraumatic Stress Disorder (PTSD) and Dissociative Identity Disorder (DID). Furthermore, James also suffered from interpersonal difficulties and anger outbursts.

Amy, a White female, was 26 years old when therapy started. She works a blue-collar temp-job, which does not line up with her university degree. Similar to James’ background, she has a history of childhood physical and psychological abuse perpetrated by her father, while her mother remained a passive witness. Amy met the criteria of Major Depressive Disorder. There were no other axis-I or axis-II disorders diagnosed.

Pam, a White female, was 33 years old. She has a graduate degree, but was unemployed when she entered treatment. She had a history of childhood physical and psychological abuse perpetrated by her mother, while her father remained a passive witness. Pam met the criteria of recurrent seasonal Major Depressive Disorder, Agoraphobia and Body Dysmorphic Disorder. Whereas James and Amy had no prior treatment history, Pam had been taking antidepressant and anti-epileptic medication for some decades and had been hospitalized for 3 months because of suicidal ideations 3 years prior to treatment.

Table 1 provides basic descriptive information about the cases and their treatment outcome. Both James’ and Amy’s scores on the self-report outcome measures suggested reliable clinical change, as measured by the Reliable Change Index (RCI, Jacobson and Truax, 1991), for depressive complaints, overall symptom burden and interpersonal difficulties. Pam’s scores suggest no change for depressive complaints and interpersonal difficulties and even reliable clinical deterioration for overall symptom burden. After treatment termination, however, Pam’s scores on the outcome measures showed a decreasing trend and 2 years after treatment ended clinical significant improvement was achieved on the BDI-II (RCI = −4.61, <−1.96, *p* < 0.05).

**Table 1:** Basic descriptive information and outcome for all three patients.

	James			Amy			Pam		
Age	23			26			33		
Trauma history	Childhood physical abuse			Childhood physical abuse Childhood psychological abuse			Childhood physical abuse Childhood psychological abuse		
Diagnosis	Posttraumatic stress disorder Dissociative identity disorder			Major depressive disorder			Major depressive disorder Agoraphobia Body dysmorphic disorder		
Outcome	Pre	Post	RCI	Pre	Post	RCI	Pre	Post	RCI
BDI-II	37	1	(RCI = −7.55 [<−1.96], *p* < 0.05)^*^	30	2	(RCI = −5.74 [<−1.96], *p* < 0.05)^*^	36	44	(RCI = 1.68, *p* > 0.05)
SCL-90-R	277	91	(RCI = −12.33 [<−1.96], *p* < 0.05)^*^	188	118	(RCI = −4.59 [<−1.96], *p* < 0.05)^*^	231	261	(RCI = 1.97 [>1.96], *p* < 0.05)^**^
IIP-32	49	7	(RCI = −8.65 [<−1.96], *p* < 0.05)^*^	57	8	(RCI = −10.01 [<−1.96], *p* < 0.05)^*^	57	68	(RCI = 1.65, *p* > 0.05)

*RCI, reliable change index; BDI-II, Beck depression inventory; SCL-90-R, symptom checklist; IIP-32, inventory of interpersonal problems*.

* *Reliable clinical change*;

** *Reliable clinical deterioration*.

#### Therapists

James’ therapist was a White male, who, when therapy started, was 30 years old and had 4 years of clinical experience. Amy’s and Pam’s therapists were White females, 30 years old with 7 years of clinical experience and 32 years old with 8 years of clinical experience when treatment started, respectively. All therapists were formally trained in Psychoanalytic Therapy. James’ therapist had a PhD in clinical psychology and Amy’s and Pam’s therapists received additional training in Short Term Psychodynamic Psychotherapy (STPP, Luborsky, 1984; Leichsenring and Schauenburg, 2014) and received biweekly group supervision (Meganck et al., 2017). James’ treatment consisted of 41 sessions of psychodynamic therapy. Amy and Pam each received 20 sessions of treatment.

### Instruments

#### Quantitative Measures

*The Beck Depression Inventory-II* (BDI-II, Beck et al., 1996) is a 21-items self-report questionnaire used to assess depression severity. *The Symptom Checklist* (SCL-90-R, Derogatis, 1992) is a 90-items self-report questionnaire administered to assess psychical and physical symptoms on nine dimensions (i.e., somatization, obsessive-compulsive, depression, anxiety, hostility, phobic anxiety, paranoid ideation, and psychoticism). *The Inventory of Interpersonal Problems-32* (IIP-32, Horowitz et al., 2000) is a self-report questionnaire to assess interpersonal functioning. The IIP-32 consists of 32 items measured on a 5-point Likert scale with response options ranging from “Not at all” to “Extremely.” The Dutch translation of the IIP-32 has good psychometric qualities and has been validated for the use of diagnostic and research purposes (Vanheule et al., 2006). *The Working Alliance Inventory* (WAI-12, Horvath and Greenberg, 1989) is self-report questionnaire aimed to assess the quality of the therapeutic relationship *via* 12 items measured on a 5-point Likert scale (1 = seldom or never, 5 = always). The Dutch translation of the WAV-12 was validated by Stinckens et al. (2009).

#### Structured and Semi-structured Interviews

*The Clinical Diagnostic Interview* (CDI, Westen, 2006) is a semi-structured narrative-based interview that assesses a broad range of intra- and inter-personal characteristics. This interview allows for an in-depth understanding of important past and current relationships that appear in the story of the patient (e.g., “How would you describe your relationship with your mother/father/partner/…?” or “Can you describe a specific situation or confrontation with him/her that typifies your relationship?”). *The Structured Clinical Interview for DSM-IV* (SCID) is a structured interview to determine DSM-IV axis I disorders (SCID-I, First et al., 2002) and DSM-IV axis II personality disorders (SCID-II, First et al., 1997). The Client Change Interview (CCI, Elliott et al., 2001) is a semi-structured interview assessing therapeutic change with explicit attention to changes in interpersonal relationships and the experience of the therapeutic relationship.

### Procedures

We selected our cases on the basis of two inclusion criteria, namely (1) a history of childhood trauma, and (2) therapy focuses on (working through) interpersonal difficulties. The cases were selected from the larger research projects (Single Case Studies, Ghent Psychotherapy Study; Meganck et al., 2017) conducted at the Department of Psychoanalysis and Clinical Consulting, Ghent University. The ethical committee of the Ghent University Hospital provided positive ethical advice on these larger process-outcome studies (EC/2015/0085; B670201318127) and informed consents were obtained from all participants.

The extensive data collection in these projects makes rigorous and systematic outcome and process studies possible (Dattilio et al., 2010). Below, we only mention the measures used in our research. The diagnostic procedure included the administration of the SCID-I and -II (First et al., 1997, 2002) and the Clinical Diagnostic Interview (CDI; Westen, 2006). These interviews were audiotaped and transcribed. During therapy, all sessions were audiotaped and transcribed according to preset standards.

In order to map interpersonal difficulties, comorbid symptoms, and general well-being, we administered the IIP-32 (Horowitz et al., 2000), the BDI-II (Beck et al., 1996), and the SCL-90 (Derogatis, 1992) pre- and post-treatment, every fourth session and at 3-month, 6-month, 1-year, and 2-year follow up. After every fourth session, the patient also had to fill out the WAI-12 (Horvath and Greenberg, 1989) to assess the therapeutic relationship. The CCI (Elliott et al., 2001) was administered peri- and post-treatment, as well as at 3-month, 6-month, 1-year, and 2-year follow up.

To investigate the nature and change in interpersonal patterns, we used *the Core Conflictual Relationship Theme method* (Luborsky and Crits-Christoph, 1998). The CCRT maps three dimensions of people’s relationships to others: the subjective wishes with which one enters interpersonal relations (W), one’s own personal appraisal of how the other interacts and responds to these wishes (RO) and the characteristic reactions of the self to this other (RS). To map the dominant CCRTs and the changes therein throughout therapy, two researchers conducted the CCRT method on narratives derived from the transcribed therapy sessions at the beginning (session 1 through 4), middle (session 9 through 12), and end (sessions 17 through 20) of treatment. Because in James’ case, there was no set time-limit, the investigated therapy sessions differ (two intake meetings and the first two therapy sessions, session 20 through 22 and session 37 through 41 to study the CCRT at the beginning, middle, and end of therapy, respectively). However, because we conducted the CCRT method in this case also at the beginning, middle, and end of therapy, we ensured the comparability of the three cases. The CCRT method starts with selecting a minimum of seven relationship episodes (REs) within the narrative material. REs are relatively discrete episodes in which a person speaks about relationships with others. The CCRT method then maps three dimensions of people’s allying with others: the subjective wishes (W), the response of the other (RO), and the characteristic reactions of the self to this other (RS). These components were rated separately by two researchers using the Standard Categories (Edition 2) provided by the CCRT manual, which includes 35 Ws, 30 ROs, and 31 RSs. *Via* consecutive consensus meetings, we systematized our research process. Consensus on the frequency of each component was achieved through detailed discussion and the final frequency with which each category occurred across the REs was computed to provide the dominant CCRT.

In the cases of Amy and Pam^2^, we investigated the formation of the therapeutic alliance *via* a quantitative investigation of the evolution of the WAI-12 scores and a qualitative investigation of recurrent patterns *via* a *thematic analysis* (Brown and Clarke, 2006). Further, we applied the *Penn Adherence/Competence Scale for SE Dynamic Psychotherapy* (PACS-SE, Barber and Crits-Christoph, 1996) to assess the frequency of different therapeutic techniques. The scale consists of nine items assessing general techniques (e.g., “The therapist encourages the patient to explore the personal meaning of an event or feeling”), nine items assessing supportive techniques (e.g., “The therapist conveys a sense of respect, understanding and acceptance to the patient.”), and 27 items assessing expressive interventions (e.g., “The therapist focuses attention on similarities among the patient’s past and present relationships”). All therapists’ interventions – except “mhm,” which was considered a neutral intervention – were rated as general, supportive or expressive by two researchers, independent from each other. Through consecutive meetings, consensus was achieved (Jackson et al., 2011) and the frequencies per technique were computed for every session. Overall, we used consensus procedures to systematize our research with triangulation over researchers, methods, and instruments (Jackson et al., 2011), which allowed comparison and transferability across cases (e.g., Levitt et al., 2018).

Finally, we synthesized our research findings using principles of qualitative metasynthesis (e.g., Iwakabe and Gazzola, 2009). This approach allowed us to identify cross-case similarities and dissimilarities in terms of (changes in) interpersonal dynamics in cases with a complex trauma history and enabled us to explore possible alternative explanations for unique or distinctive features within the individual cases.

## Results

### The Nature of Interpersonal Patterns at the Beginning of Treatment

To integrate our findings, we aggregated the results of the three cases per phase (beginning, middle, and end of therapy). Table 2 provides an overview of the accumulated results. At the beginning of treatment, the wish (W) “to be respected” and “to not be hurt” prevailed in all three cases. Others (RO) were perceived as “rejecting,” “not understanding,” “disrespectful,” and “distant,” which rendered our subjects (RS) feeling “angry.” Luborsky and Crits-Christoph (1998) make a distinction between positive and negative ROs and RSs. It is clear that in all three cases, the reactions from others, as well as their own reactions, are perceived in a negative way. Furthermore, the reactions from our subjects are not only negatively connotated but also demonstrate a passive position and a lack of agency (e.g., “am not open,” “am dependent,” “am helpless”). In this context, it is important to note that the anger our subjects felt was not expressed toward others. Moreover, the helpful attitude that both James and Pam expressed could be seen as passive or dependent reactions because their behavior did not correspond with what they longed for in relation to others (W “to be respected,” “to be loved,” “to be helped,”) and rather showed a submissive compliance in order to protect themselves (W “to not get hurt,” “to avoid conflict”) in the face of the anticipated “rejection” and “disrespect.”

**Table 2:** The main CCRT components over three cases at the beginning, middle, and end of treatment.

	#	W	RO	RS
Phase 1	3–30	To be respected (3–12)/to not be hurt (3–10)/to be loved (2–6)/to be helped (2–5)/to be accepted (2–4)	Are rejecting (3–21)/are not understanding (3–13)/are distant (3–11)/do not respect me (3–7)/are controlling (2–11)/are bad (2–6)/are not trustworthy (2–5)/are angry (2–4)	Feel angry (3–12)/am not open (2–18)/feel anxious (2–14)/am dependent (2–11)/feel disappointed (2–10)/feel depressed (2–6)/am helpless (2–6)/am helpful (2–4)
Phase 2	3–30	To be respected (3–19)/to be accepted (2–10)/to be open (2–7)/to be liked (2–6)/to be understood (2–6)	*Respect me* (2–9)/are distant (2–9)/are rejecting (2–9)/are not understanding (2–7)/do not respect me (2–7)/are not trustworthy (2–6)/*are open* (2–5)/are controlling(2–5)/*are understanding* (2–4)	Am uncertain (3–11)/*feel respected* (2–8)/*feel comfortable* (2–8)/*am open* (2–8)/feel angry (2–8)/feel disappointed (2–7)/feel anxious (2–7)/am helpless (2–7)/*feel happy* (2–6)/*am independent* (2–6)
Phase 3	3–35	To be respected (3–22)/to be accepted (2–12)/to be understood (2–10)/to assert myself (2–9)/to be liked (2–8)/to be opened up to (2–6)/to be open (2–6)/to have control over others (2–5)/to be helped (2–5)	Do not respect me (3–11)/*respect me* (3–8)/are rejecting (2–15)/are controlling (2–10)/*are open* (2–8)/are unhelpful (2–8)/are not understanding (2–8)/oppose me (2–7)/*are understanding* (2–6)/are bad (2–5)/*are accepting* (2–5)/*are cooperative* (2–5)/are out of control (2–4)	Feel disappointed (3–14)/feel angry (3–12)/*feel self-confident* (3–11)/oppose others (2–8)/am helpless (2–7)/*feel respected* (2–6)/*am self-controlled* (2–5)/am uncertain (2–5)/*feel accepted* (2–4)/am controlling (2–4)/*feel happy* (2–4)

*Phase 1, beginning of treatment; Phase 2, middle of treatment; Phase 3, end of treatment; #, number of cases − number of relationship episodes; W, wish; RO, response of other; RS, response of self; (x-y), number of cases − number of relationship episodes; italic, positive RO or RS*.

On the level of the dominant wish, our results correspond with studies demonstrating the prevalence of the wish “to be close and accepted” (Okey et al., 2000) or “to be loved and understood” (Chance et al., 2000). The contrasting wish “to oppose others,” “hurt others” or “control others” (e.g., Frueh et al., 2001; Drapeau and Perry, 2009) could only explicitly be observed in Amy’s case. However, these wishes were strongly interconnected with the wish “to not be hurt.” Our results suggest that certain wishes, such as “to oppose others,” “to control others,” “to not be hurt,” and “to avoid conflict,” are actually subordinate to the wishes “to be loved,” “to be respected,” and “to be accepted” and are formulated only because the subjects anticipate these latter wishes to be frustrated by others’ reactions of ignorance (RO “are not understanding,” “are distant”) and contempt (RO “are rejecting,” “don’t respect me”). In the broader field of studies concerning interpersonal patterns related to psychopathology in several patient groups, it has been found that the most common wish is to be close to others and to be accepted (e.g., Wilczek et al., 2010).

Regarding the dominant (perceived) response of others, we found strong support for the prevalent perception of others being “rejecting” (Chance et al., 2000; Okey et al., 2000)^3^. The perception of others as “controlling” (Drapeau and Perry, 2009) appeared explicitly in the cases of Amy and Pam, whereas in James’ case, it appeared more implicitly in his submissive reaction (RS “am dependent”) toward others. We further found support for the perception of others as malignant (RO “are bad,” “are angry,” e.g., Tummala-Narra et al., 2012) and the prevalence of mistrust (RO “are not trustworthy,” e.g., Ebert and Dyck, 2004). Whereas in the literature, feelings of mistrust are put forward as the core characteristic feature of childhood trauma (e.g., Herman, 1992; Pearlman and Courtois, 2005), our results warrant to also take others’ “misunderstanding,” “distance,” and “disrespectfulness” into consideration, as these components explicitly accrued in all three cases.

Finally, we found support for patients’ own reactions of feeling “depressed,” “disappointed” (Chance et al., 2000; Okey et al., 2000), “anxious,” and “helpless” (e.g., Ebert and Dyck, 2004; Tummala-Narra et al., 2012) and the tendency to keep silent (RS “am not open,” e.g., Cook et al., 2004). On the basis of the literature, we would also have expected feelings of shame, guilt, and self-blame to be dominant (e.g., Cloitre et al., 2009). However, we only found minor indications of the prevalence of these components as “feeling guilty” and “feeling ashamed” only accrued in the cases of Pam and Amy, respectively. What stood out in our results was the feeling of “anger” toward others. Whereas in the literature, this has been described as active hostility and aggressive behavior (e.g., Frueh et al., 2001; Cloitre et al., 2009), again, our results suggest that the patients’ anger was not expressed overtly. Within the broader perspective of our findings, the inhibition of anger feelings can be understood as a defense strategy because expressing anger might “threaten the very hand that feeds” (Blatt, 2004), whereas refraining from anger might aid the pursuit for nurturance.

The narratives of our three cases reveal a history of childhood maltreatment, both in terms of physical and psychological abuse. When James was a child, he (passively) obeyed his father’s demands, notwithstanding his interior anger and disappointment, out of fear for retaliation. Amy also feared her fathers’ anger outbursts and avoided them by not expressing her (anger) emotions. Finally, Pam tried to avoid the feared conflicts with her parents, especially with her mother, by retaining a passive stance and keeping silent. What stands out in all three cases, is how they *feared* their parent(s) and *tried to avoid confrontation* by taking up a *passive position* toward them and showing a *reluctance to express themselves*. James, Amy, and Pam stated that they were *feeling* angry at the time of the abuse, but in no way were able to *express* this anger.

The adverse circumstances in which the subjects were brought up, forced them to create schemes to understand and adapt to the dysfunctional situation, which form a deeply engrained internal working model, which color the subjects’ later relationships (e.g., Walsh et al., 2010). James, for instance, submissively obeyed the anger provoking demands of his girlfriend out of fear of rejection, while strongly aspiring a loving and close relationship. Amy, on her part, strongly wished to be able to express her desires and emotions freely, but prevented herself from doing so out of fear of receiving critical and rejecting reactions. Pam then, in her adult love and work relationships, did not open up, despite wanting to assert herself, because she wanted to protect herself from the anticipated criticism of others. A general pattern we can distill from these subjects’ singular narratives, is the *inability to express desires and emotions* to *avoid anticipated negative, rejecting reactions* from others. We thus see a clear resemblance between the reaction patterns in childhood and adulthood. This provides support for the assertion that childhood adverse experiences lead to certain relational patterns which influence and manifest themselves in adult relationships (e.g., Gleiser et al., 2008).

All in all, our findings show that the relationship between exposure to childhood trauma and the (interpersonal) consequences is neither universal (i.e., a one-to-one relationship with identical reactions in every case) nor absolute relativistic (i.e., the relationship between event and reaction depends on too many context-specific variables to extract certain patterns across cases). Instead, the relationship between the exposure to traumatic events and traumatic reactions can be understood *via* the principle of *universalism without uniformity* (Soenens et al., 2015), meaning that, notwithstanding every person has a unique response to the exposure to childhood adversities, certain patterns recur across cases. It is interesting to note that the negative (perceived) response of others was most similar in our cases, which frustrated their overall wish for closeness. In this context, more important than the tendency to stay close to others or to keep distance (e.g., Cook et al., 2004; Cloitre et al., 2009), is the tendency to *avoid* the negative responses of others, be it by either actually keeping distance or by resorting to submissive compliance. In other words, they are all passively subjected to the other without any agency to pursue their own desires. This has important implications for therapy, as it is with this preoccupation to manage the response of others that the subject enters therapy.

### Changes in the Core Conflictual Relationship Theme Throughout Treatment

As Table 1 illustrates, the wish “to be respected” remains dominant in all three cases in the middle of treatment. Likewise, the wishes “to be accepted” and “to be liked” (in phase 1 “to be loved”) prevail. What stands out is that the wish “to not be hurt,” which was present in all three cases in phase 1, is no longer on the forefront and is only mentioned twice by Pam. Next to that, we see that the wish “to be open” also emerged in Pam’s case. An overall tendency seems to be that our subjects articulate their desire no longer (James and Amy) or less (Pam) in a passive voice, meaning they no longer formulate what they aspire from relationships in a negative (“I don’t want to…”), but rather in an active way (“I want to…”).

There is also a notable shift in the response of others and the response of self. Whereas in phase 1, there were, in general, only negative ROs; in phase 2, there are also positive ROs with the exception of Pam’s case. Especially the ROs “respect me” and “are understanding” stand out in the cases of James and Amy because these responses satisfy the wish “to be respected” and “to be understood.” In accordance, James and Amy express positive RSs, such as “feel respected,” “feel comfortable,” and “am open,” whereas in Pam’s case, there are only negative RSs. It thus seems that the perceived response of others and the way that response endorses the main wish strongly influences the way the subjects view and position themselves in relationships. This could imply that they are still rather subject to the response of the other. This is perhaps most clearly illustrated in James’ case. James was surprised by the positive and encouraging reactions of others with regards to his recent suicide attempt and their positive reactions made him feel “loved” and “respected.” The relationship episodes concerning his ex-girlfriend, however, showed that a negative reaction on her part still provoked a negative response in James.

Finally, in phase 2, we see that the RS “am uncertain” is the most prevalent and occurs in all three cases. In Pam’s case, ambivalence ensued when she described the wish to be more open toward others. Amy articulated uncertainty to continue her relationship, and James felt torn between feelings of love and anger toward his ex-girlfriend Rebecca and whether or not to move forward in relation to his friend Holly. It thus seems that, notwithstanding the influential nature of the others’ responses described above, our subjects take a more active position and begin to interrogate their position vis-à-vis important others in their lives. We could assume that this is part of the process of change that is ensuing.

At the end of treatment, Table 1 illustrates that the dominant wish “to be respected” holds up in all three cases. This corresponds with the overall tendency in therapy that wishes do not particularly change (Luborsky and Crits-Christoph, 1998; Wiseman and Tishby, 2017). However, the trend we observed in phase 2, namely that the wishes were being formulated in a more active voice, sustained until the end of treatment as evidenced by the wishes “to assert myself,” “to be open,” and “to have control over others.” Distinctive here is that there is a wide variety of possible positive and negative responses of others and self in all three cases. James, Amy, and Pam recount situations in which others were either perceived as “disrespectful” or “respectful.” So, in all cases, in some instances, their main wish “to be respected” was fulfilled. Interestingly, this pattern does not seem to automatically correspond with “feeling respected,” especially in Pam’s case in which this RS was not accounted for. Correspondingly, the negative responses of others did not always provoke a negative reaction in our subjects anymore. James, for instance, upheld a positive position, regardless of whether others initially were perceived as uncooperative. Similarly, Amy embraced the continued negativity of others and continued to stand up for herself, notwithstanding sometimes feeling helpless or uncertain. Overall, the available ROs and RSs suggest very diverse interactional patterns between our subjects and important others. Therefore, we cannot formulate a characteristic structure of the CCRT at the end of treatment. This suggests that our subjects are no longer trapped in a fixed template of interacting with others. Moreover, the observation that all subjects expressed more self-confidence in relationships conveys the impression that at the end of treatment, they were able to take a more active and dynamic stance with a sense of agency and control. Despite the fact that the outcome was not unequivocally positive, it thus appears that change in the CCRT components was established in all three cases^4^.

### The Process Component of the Core Conflictual Relationship Theme Throughout Treatment

#### The Formation of the Therapeutic Relationship

To integrate our findings, we systematically compared the quantitative and qualitative data of the process of change in the cases of Amy and Pam. The most curious and unexpected observation was that in both cases the therapeutic relationship seemed to be readily established. In Amy and Pam’s cases, the quantitative analysis of the WAI-12 (Horvath and Greenberg, 1989) suggested that feelings of mutual trust (bond scale), consensus on treatment objectives (goal scale), and ways to accomplish them (task scale) were achieved early in treatment. Correspondingly, both Amy and Pam commented rather positively on their relationship vis-à-vis the therapist in the CCIs (Elliott et al., 2001). Pam, for instance, recounted that the therapist was friendly and professional and Amy praised the therapist’s neutrality, acknowledgment, and empowerment. However, in both cases, these results should not be taken at face value. Especially in Pam’s case, we could observe that a fundamental feeling of trust was lacking at the beginning of treatment. This was evinced by the fact that Pam relied on the professional confidentiality of her therapist to ensure discretion and that she did not dare to communicate her distress in treatment. We linked this lack of fundamental trust to Pam’s general stance in relationships, i.e., her CCRT at the beginning of treatment, in which she would be quiet and apprehensive in interactions because she expected others to be unreliable and deceitful. Perhaps to a lesser extent, this also occurred in Amy’s case. Amy feared being labeled crazy whenever she would express herself openly. This pattern repeated itself in treatment *via* a more rational presence and preparing the therapist that what was about to come out of her mouth might sound crazy. These observations correspond with the idea that certain elements of the therapeutic relationship cannot be accessed or assessed *via* self-report measures because of certain underlying dynamics that are unconsciously influencing the exchanges between patient and therapist (e.g., Waldinger et al., 2003).

The core interpersonal patterns implicitly manifest and repeat themselves in treatment and therefore automatically impact the therapeutic relationship. Because Pam and Amy unconsciously expected a certain negative reaction from their therapists, they were not able to express themselves openly at the beginning of treatment. In other words, the therapeutic environment was not inherently seen as a safe environment, notwithstanding the objective qualification of the therapeutic relationship as satisfactory. In this way, we found confirmation for the idea that building a trusting relationship with patients with a childhood trauma background might be a precarious task (e.g., Ebert and Dyck, 2004). However, contrary to the literature in which it appears that a lack of trust is manifested rather overtly in treatment and resolutely warrants attention to the formation of the therapeutic alliance (e.g., Pearlman and Courtois, 2005), our results indicate that issues of trust might remain obscure. Therefore, we postulate that therapists should always be wary of the nature of the therapeutic relationship. In this, it is not only a matter of checking the overt qualities of the relationship, but, more importantly, to be aware of the dynamics underlying the interpersonal exchange.

No straightforward recommendations can be made in order to guarantee a sustainable therapeutic relationship because these underlying dynamics differ from person to person and should be reviewed case by case. Our CCRT results revealed some commonalities over cases, which allow a more general rule of thumb, namely to avoid getting caught up in a repetition of the CCRT by providing a different response and thereby constituting a different other for the patient. Amy entered treatment with the same anticipation of being labeled crazy she had in other relationships. In contrast to what she would expect from others, the therapist exerted a neutral, acknowledging, and empowering attitude. As a result, Amy did not have to fear criticism or rejection, thereby she did not have to avoid these reactions by purposefully adjusting her own conduct and was able to explore and work through her interpersonal issues. Pam showed to be very introverted and cautious at the beginning of treatment, in accordance with her general tendency to avoid someone betraying her trust. It was only when the therapist actively communicated her genuine interest and appreciation and restrained from any authoritarian whim that Pam was able to open up more safely.

As our results show, our subjects anticipated others to be “rejecting,” “not understanding,” “distant,” and “disrespectful,” which, of course, warrant the general recommendation of providing warmth and acceptance in treatment (e.g., Wampold, 2007). However, these non-specific therapist factors do not suffice, as the determining factor is the patient’s perception of the therapist’s genuineness and authenticity (Gleiser et al., 2008; Lawson et al., 2013). To illustrate, Pam readily described her therapist as a friendly person, showcasing that the non-specific therapist factors were in place. Nevertheless, as outlined above, this certainly was insufficient for the formation of a safe environment. At the end of treatment, Pam declared that the therapist felt familiar and safe, which demonstrates a more fundamental connection between them. This change was connected to the therapist’s decision to alter her treatment to a more supportive approach in order to create a better fit with Pam’s needs. This was also explicitly cited by Amy when she mentioned that her therapist’s “way of approaching things corresponded to [her] needs.” Amy stressed the importance of her therapist’s neutrality, reassurance, and empowerment, which also might be considered non-specific therapist factors. She demonstrated the importance of her therapist’s neutrality in situations where she would normally expect an accusing finger, whereas she commented on the therapist’s reassuring and empowering statements on very particular instances in which Amy felt uncertain about herself. This suggests that the therapist tailored her therapeutic approach on the basis of her knowledge about Amy’s interpersonal sensitivities, thus adapting her interventions quite specifically to Amy’s case. These findings illustrate the importance of therapists’ responsiveness in treatment, which means that therapists are attentive to patients’ (changing) needs and resources and appropriately adapt their interventions accordingly (Stiles, 1998). This does not only apply to the overt speech and behavior of patients. Our findings show the importance of those dialectical moments in which the therapists conveyed a deeper understanding and attuned their interventions to the underlying dynamics or CCRT components that influence the therapeutic exchange.

#### Therapist Interventions Throughout Treatment

Our results indicate that the therapeutic relationship was seemingly established quite easily. From this observation, we could already suspect that our results would fail to meet our expectation that the beginning of treatment would be dominated by supportive interventions to establish a safe therapeutic relationship, followed by more expressive interventions, which focus on working through the CCRT. In our pilot study of James’ case, in which we did not study the therapeutic interventions in a systematic way, we already observed that the therapist implemented expressive interventions from the outset, without any specific or special efforts to build the therapeutic relationship. In this context, the use of supportive techniques served the purpose of maintaining the already established relationship. Also in Pam and Amy’s cases, in which the therapists’ interventions were systematically studied, we could not find the expected sequence of supportive and expressive interventions. Table 2 shows the total distribution of supportive, expressive, and general interventions throughout therapy. In Amy’s case, we saw that expressive interventions were more frequent in all sessions, with the exception for sessions 1, 12, and 18. On the other hand, in Pam’s case, the sequence of supportive and expressive interventions showed a more erratic sequence, with alternatingly more supportive and expressive interventions throughout treatment. The therapeutic processes of Amy and Pam thus demonstrate very distinct treatment trajectories. Below, we discuss which conclusions can be drawn from the commonalities and dissimilarities between cases.

A first notable difference between the therapy processes of Amy and Pam is the number of interventions used throughout the sessions. Pam’s therapist used twice as many interventions per sessions (*M* = 119) in comparison to Amy’s therapist (*M* = 55). In order to facilitate Pam’s speech, the therapist mainly used a large number of general interventions, including neutral questions and small reiterations. Amy’s therapist also used a large number of general interventions by ways of encouraging further speech.

Second, common in both cases is the general preponderance of expressive interventions with on average 17 and 36 expressive interventions per session in Amy’s and Pam’s case, respectively. At the beginning of treatment, the two therapists used expressive interventions to gather information about their patients’ interpersonal relations and the position of the different people involved. Further, in both cases, supportive interventions were stacked at the end of the first treatment sessions and were used to convey a commitment to their work together.

Third, in both cases, we saw a shift in the purpose of the expressive interventions the therapists used. Whereas at the beginning of treatment, the expressive interventions were used to gather information about interpersonal issues, they were applied gradually more with the aim to work through interpersonal difficulties. This was made possible by the fact that both Amy and Pam recognized and acknowledged their own position in relationships, i.e., they conveyed a sense of understanding concerning their CCRT and both expressed a wish to make a change. However, there is a noticeable difference in the way Amy and Pam responded to these interventions. Amy was able to elaborate on her (RS) and significant others’ (RO) general position in relationships and started to explore how these components influenced very specific interpersonal encounters. The therapist supported this working through *via* additional supportive interventions. Pam, on the other hand, was not able to safely explore and work through her interpersonal issues. Instead, we saw a worsening of her depressive complaints and an increase in distress. We saw that the treatment took a radical turn after the therapist discussed Pam’s situation in supervision. The therapist shifted her attention from the (trauma-related) interpersonal difficulties to issues Pam encountered in her everyday life, such as working toward a daily structure and dealing with bodily distress. Moreover, she used a greater number of supportive interventions. Here, the therapist not only used supportive interventions to convey her genuine interest and honest commitment, but also – in parallel with Amy’s case – to deliver expressive interventions in a more supportive way by stacking an expressive intervention on a supportive one. It thus seems that certain interpretations might be digested more easily when delivered with care and support. This becomes even more apparent when we take into consideration that Amy stressed the importance of her therapist’s supportive attitude, notwithstanding the latter did not use a tangible number of supportive interventions. We noted that several more general and non-verbal gestures (e.g., changes in tone of voice, laughter) added to the supportive atmosphere. Similarly, in Pam’s case, general interventions helped build up a safe environment. Amy’s and Pam’s cases thus show that the experience of a supportive environment can depend on very different things, regardless of the number of supportive interventions that specifically aim to foster such an environment.

## Discussion

Systematic single case studies have been endorsed because of their in-depth and context-rich scrutiny. However, “[o]ne observation or one case offers only a small piece of evidence, but repeated observation […] across a series of cases provides a way of constructing a database of evidence on which clinical theory can be built.” (Dattilio et al., 2010, p. 436). In this way, from the results of our case comparison study, we can draw several clinical implications and recommendations.

Our first recommendation is associated with our observation that therapists should be wary about the impact their interventions have on their patients. It has been suggested in the literature that therapists are notoriously bad at estimating the effects of their interventions (e.g., Hatfield et al., 2010). In Pam’s case, we saw an interconnection between symptom severity and the treatment process. The evolution in her symptoms followed a U-shaped curve. Symptoms worsened up until the middle of treatment, after which they steadily started to decline. We could connect this development in symptom burden to the number of expressive and supportive interventions the therapist used throughout treatment. An excessive number of expressive interventions was accompanied by a worsening of Pam’s condition, whereas the mid-course correction to more supportive interventions went together with an improvement in symptom severity. However, although Pam reported feeling better in the CCIs, these changes were not captured in the self-report questionnaires up until follow-up. Notwithstanding our results are formed on the basis of longitudinal observations, it is therefore premature to draw any firm (causal) conclusions from this. However, our results do indicate the importance of tracking patients’ complaints and symptomatic burden. Here, it does not suffice to assess symptoms and other difficulties *via* self-report questionnaires. As we have seen in the context of the therapeutic alliance, there might be a vast difference between the patient’s conscious estimation of his/her condition and the underlying processes.

Our second recommendation, which is closely related to the first, is to apply treatment strategies amenably and attuned to the patient’s needs (e.g., Beutler et al., 2016). In general, therapists are advised to intervene with appropriate responsiveness, meaning that their interventions are accustomed to the patient’s needs and resources (Stiles, 1998). This seems self-evident, but research has shown that therapists sometimes persistently hold on to their treatment regimens even when the patient does not respond in a foreseen or benevolent way (Castonguay et al., 2010). As the different responses of Amy and Pam to expressive interventions indicate, this does not mean that the interventions themselves are faulty; yet that there is a mismatch between patient, therapist, and treatment interventions. This point is particularly salient given the strenuous discussion about appropriate treatment strategies in the complex trauma literature. There is a disagreement in the field regarding whether or not a phase-based treatment approach is necessary in order to appropriately treat patients with a childhood trauma background (e.g., Resick et al., 2012). Our results suggest that this is not an either/or decision and that we should consider taking into account the mechanisms of change (Kazdin, 2009) to make any sound recommendations. In the case of Amy, we have noted that Amy responded particularly well to expressive interventions, which lines up with treatment approaches that protest against the use of initial stabilization (e.g., Wagemans et al., 2018). In contrast, in Pam’s case, we argued that a straightforward approach on traumatic contents was non-profitable and instead, her case warranted a supportive, stabilizing approach (e.g., Jepsen et al., 2013). In both cases, however, we readily articulated our reservations about such a dichotomous vantagepoint. Instead, we argued for a more dimensional or flexible approach in which for every individual patient the amounts and appropriateness of supportive versus expressive interventions has to be weighted and balanced in light of the broader context and narrative of the case. Moreover, therapy is a fluid situation and the interactions between therapist and patient change dynamically as a function of numerous factors inside (e.g., increased self-understanding of the patient, growing therapeutic alliance) and outside (e.g., social support, life events) the therapy (Polkinghorne, 1999). Therefore, a continuous back-and-forth between monitoring the patient’s overall condition and the practical customization of therapist interventions is warranted (Stiles, 1998; Polkinghorne, 1999).

Third, we want to point out the importance of discussing (difficult) patients in supervision. Studies have revealed that therapists are not always able to make a fair estimation of the (negative) effects therapy produces (e.g., Hatfield et al., 2010). Such an evaluation of the treatment process seems, however, necessary because it could determine whether a change in the treatment approach is recommended. The benefits of psychotherapy supervision have been widely acknowledged, not only for training psychologists; it is also often endorsed as a general prerequisite for the practice of psychotherapy (e.g., Luborsky, 1984; Dulsster and Vanheule, 2019). Also in the complex trauma literature, supervision has been put forward as a valuable way to recognize and understand therapists’ actions and reactions in treatment (e.g., Pearlman and Courtois, 2005). This is further illustrated in Pam’s case in which the therapist discussed the case of Pam in supervision and, accordingly, changed her treatment approach. Particularly interesting here was that the therapist asked herself whether it was her own desire, rather than Pam’s, to work through the traumatic contents and explore the traumatic nature of Pam’s relationship with her mother. In other words, the therapist questioned the universal treatment guideline in the trauma literature to focus on the traumatic contents (e.g., American Psychological Association, 2017), but also the central focus of working through interpersonal difficulties in supportive-expressive psychodynamic therapy (e.g., Luborsky, 1984). Therefore, this question pertinently demonstrates the potential of being blindsided by theoretical convictions or clinical preferences (Castonguay et al., 2010). Next to that, the outcome of the supervision illustrates how it can lead to new perspectives on the treatment process of a particular patient. In Pam’s case, we saw that after the supervision, the therapist applied much more supportive techniques and shifted the focus of the treatment to the more pressing issues in Pam’s daily life, which gave rise to an improvement in her overall condition and well-being. A more detailed discussion of the supervision process was beyond the scope of this study. However, it would be interesting to investigate supervision sessions in greater detail and in a more systematic way in order to create a fuller understanding of how supervision can aid therapists in their clinical work.

Fourth, we want to comment on the importance of facilitating patients’ agency in treatment. In our studies, we have seen that allowing the patient agency was pivotal. For Amy, being able to steer the conversation contributed to feeling safe to set boundaries regarding what she felt comfortable to talk about. In Pam’s case, we have seen that the therapist eventually discontinued the tenacious focus on talking about Pam’s traumatic history and, instead, let Pam decide whether or not to talk about those delicate issues. In parallel with the importance of supervision, allowing patients agency has been put forward as a more general guideline for good practice (e.g., Levitt et al., 2016). Moreover, in the trauma literature, special emphasis has been placed on giving agency to patients (e.g., Herman, 1992). The underlying logic consists of the idea that childhood trauma victims were repeatedly placed in a passive position by their aggressors, which undermined their sense of personal entitlement and agency (Brown et al., 2012). We found that James, Pam, and Amy maintained a passive position vis-à-vis others. In this context, it is important to prevent placing the patient again in a passive position. Instead, it is better to allow the patient control over the therapeutic situation in order to enhance their sense of agency (e.g., Herman, 1992) and to prevent them from feeling subjected to the control of someone else (e.g., Liotti, 2013). In other words, as a therapist, it is recommended to refrain from a position of control in order to prevent a repetition of the (traumatic) relational experiences the patient has sustained and to allow a new relational experience from which change can ensue.

This leads us to our last clinical implication, which we have touched upon already a number of times and seems to encompass all of the above, namely to address interpersonal patterns in diagnosing and treating patients with a childhood trauma background. It is important to have insight in the dominant interpersonal patterns because a repetition of these patterns can hamper the establishment of the therapeutic relationship. Moreover, understanding the nature of core interpersonal patterns is considered necessary in order to be able to create a new relational experience for patients. Finally, we have seen that this allows patients to safely and freely express themselves in treatment, by which opportunities are created to work through interpersonal issues, to alleviate symptoms and to augment patients’ overall well-being.

In general, our results indicate that there is a strong interconnection between dominant interpersonal patterns and the process of change in the treatment of cases with a childhood trauma background. We found that deeply engrained interpersonal patterns, which are formed in relation to primary caregivers, translated into severe difficulties in interpersonal functioning in later life. Further, we saw that a new relational experience, with a therapist that constituted another other for the patient, created opportunities to revise and rework these deeply engrained interpersonal patterns, which allowed our subjects to position themselves differently in relation to themselves, others, and the world.

Notwithstanding treatment should always be considered within the context of the particular case, clinicians can draw from this case comparison study to make more effective clinical decisions (Edwards et al., 2004) and to be more attentive and responsive to the needs and resources of their own patients (Stiles, 1998). Further, our research can stimulate clinicians to integrate scientific and theoretical knowledge into practice, which offers tools to understand the psychopathology of their patients and subsequently can help shape treatment plans (Eells, 2007; Vanheule, 2017).

*Via* triangulation of researchers, resources and methods, and the use of consensus procedures (Jackson et al., 2011), we aimed to safeguard the validity and reliability of our findings. In both Amy’s and Pam’s case, we could observe several discrepant findings between our qualitative and quantitative measures. For instance, we distinguished between the objective appraisal of the therapeutic relationship *via* the WAI-12 and the underlying interpersonal dynamics which (covertly) affect the relationship. In comparing and discussing these discrepancies, our cases have provided us with some critical insights (Stiles, 2013) to understand interpersonal patterns in childhood trauma and how they influence the therapeutic process. These findings are indicative of the limitations inherent in an exclusive reliance on quantitative measures in the context of psychotherapy research and practice (Desmet, 2018).

Although we were able to discuss numerous interesting findings and formulate several theoretical and clinical implications, there are also some issues to address regarding the transferability and generalizability of our findings to other cases. Although our results confirmed that patients have quite dissimilar reactions, in terms of symptomatic burden in the aftermath of childhood trauma (e.g., van der Kolk, 2005), our sample is relatively homogeneous in terms of symptom severity. Childhood trauma has often been associated with more severe personality disruptions, such as borderline personality disorder and manifest affect-regulation problems (e.g., Herman, 1992). A degree of homogeneity is necessary in order to be able to draw valid and reliable conclusions from the comparison of cases (e.g., Iwakabe and Gazzola, 2009). However, it is necessary to stress that our implications can only be transferred to cases with a similar condition. Therefore, there is a definite need to study interpersonal patterns, the formation of the therapeutic relationship, and the therapeutic process further in non-traumatized control cases and patients with fewer internal resources and more severe characterological dysfunctions.

Further, the general problem with the CCRT is its difficulty in discriminating between different types of patient samples. In our research, this might be even more cumbersome, because all three patients also suffered from depressive symptoms, which in the literature has been associated with roughly the same CCRT components we distilled in our cases (Luborsky and Crits-Christoph, 1998; Wilczek et al., 2010). Nevertheless, our research provides an in-depth understanding of the underlying mechanisms of these CCRT components and how they are interlaced with the broader context and traumatic background of our cases. Yet, further research is necessary to investigate these mechanisms in other patient groups (for instance, cases with depressive complaints without a childhood trauma background). Further, it would be interesting to take into consideration other factors, such as personality styles. The CCRT components in the cases of James, Amy, and Pam bear a certain resemblance to the characteristics of an anaclitic personality style, as described by Blatt (2004), including dependency, a lack of assertiveness and passive obedience. However, early adversity can also be associated with an introjective personality style, which is characterized by a focus on self-definition, independency, autonomy, and achievements (Blatt, 2004). It would be interesting to study whether CCRT components differ according to personality styles and how these different interpersonal patterns influence the therapeutic process (Blatt, 2004; Meganck et al., 2017). Our main concern when selecting our sample was to ensure rich information (Patton, 2002) on interpersonal dynamics in childhood trauma. It is important to note that our results can only be understood and interpreted within the boundaries of the contexts and narratives of our cases. These limitations are inherent to case study research (e.g., Levitt et al., 2018). This means that we cannot formulate general principles that are applicable to all cases with a childhood trauma history. Nonetheless, we wish to strongly emphasize the merits of case study research and case comparison studies. They not only allow in-depth scrutiny and provide interesting insights regarding the phenomenon under study (i.e., enriching, Stiles, 2013), they also allow to refine and extend our knowledge (i.e., theory-building, Stiles, 2013). Notwithstanding case study research is laborious, time- and cost-consuming, efforts must continue to conduct and promote systematic case studies and case comparison studies in order to shed light on the mechanisms of change in treatment (e.g., Iwakabe and Gazzola, 2009; Kazdin, 2009; Dattilio et al., 2010).

## Data Availability Statement

The datasets generated for this study are available on request to the corresponding author.

## Ethics Statement

The studies involving human participants were reviewed and approved by Ethical Committee of the University Hospital of Ghent University. The patients/participants provided their written informed consent to participate in this study. Written informed consent was obtained from the individual(s) for the publication of any potentially identifiable images or data included in this article.

## Author Contributions

KV is the main author of the manuscript and contributed to the data collection, analysis, and interpretation. RM is the main reviewer and auditor of the manuscript, coordinated the data collection, and contributed to the data interpretation.

### Conflict of Interest

The authors declare that the research was conducted in the absence of any commercial or financial relationships that could be construed as a potential conflict of interest.

## Footnotes

^1^In order to guarantee the anonymity of the participants, we used pseudonyms. Moreover, all information that would lead to the identification of the patients has been removed or anonymized.

^2^We only studied the process of change in the cases of Amy and Pam because the research procedure of the Ghent Psychotherapy Study allowed a more structured investigation of the formation of the therapeutic alliance and the use of supportive and expressive interventions throughout treatment. James’ case was selected from the Single Case Studies, in which the WAI-12 was not administered.

^3^Note that the perception of others as rejecting has also been found as a dominant CCRT component in patients with major depressive disorder (e.g., Barber et al., 1995; Wilczek et al., 2010).

^4^Whereas Amy and James showed significant improvement throughout the course of therapy, Pam’s outcome scores suggested a worsening of her overall condition. Despite the minor changes we could observe in the CCRT components in the case of Pam and the improvements after treatment termination, it is interesting to note that the perseverance of CCRT components throughout treatment has been associated with negative outcome in the literature (e.g., Wilczek et al., 2010).
